# Inflammasomes and the Maintenance of Hematopoietic Homeostasis: New Perspectives and Opportunities

**DOI:** 10.3390/molecules26020309

**Published:** 2021-01-09

**Authors:** Lijing Yang, Mengjia Hu, Yukai Lu, Songling Han, Junping Wang

**Affiliations:** State Key Laboratory of Trauma, Burns and Combined Injury, Institute of Combined Injury, Chongqing Engineering Research Center for Nanomedicine, College of Preventive Medicine, Third Military Medical University, Chongqing 400038, China; yanglijing1997@sina.com (L.Y.); humengjia@tmmu.edu.cn (M.H.); luyukai@tmmu.edu.cn (Y.L.); hansongling@tmmu.edu.cn (S.H.)

**Keywords:** inflammasome, hematopoiesis, hematopoietic stem cells, hematological diseases, purinergic signaling

## Abstract

Hematopoietic stem cells (HSCs) regularly produce various blood cells throughout life via their self-renewal, proliferation, and differentiation abilities. Most HSCs remain quiescent in the bone marrow (BM) and respond in a timely manner to either physiological or pathological cues, but the underlying mechanisms remain to be further elucidated. In the past few years, accumulating evidence has highlighted an intermediate role of inflammasome activation in hematopoietic maintenance, post-hematopoietic transplantation complications, and senescence. As a cytosolic protein complex, the inflammasome participates in immune responses by generating a caspase cascade and inducing cytokine secretion. This process is generally triggered by signals from purinergic receptors that integrate extracellular stimuli such as the metabolic factor ATP via P2 receptors. Furthermore, targeted modulation/inhibition of specific inflammasomes may help to maintain/restore adequate hematopoietic homeostasis. In this review, we will first summarize the possible relationships between inflammasome activation and homeostasis based on certain interesting phenomena. The cellular and molecular mechanism by which purinergic receptors integrate extracellular cues to activate inflammasomes inside HSCs will then be described. We will also discuss the therapeutic potential of targeting inflammasomes and their components in some diseases through pharmacological or genetic strategies.

## 1. Introduction

Hematopoiesis is a dynamic and continuous process involving the production of numerous immature and mature blood cells, which mostly relies on the self-renewal, proliferation, and differentiation abilities of HSCs [1]. The maintenance of HSCs is extracellularly regulated by the BM niche, which is mainly composed of hematopoietic cells, stromal cells, adipocytes, blood vessels, and nerves [2]. Determination and trafficking of HSCs can be stimulated by a variety of juxtacrine interactions (cell–cell or cell–matrix) or paracrine interactions (via cytokines, chemokines, or growth factors) associated with the HSC niche [2,3]. Most HSCs are quiescent in the BM under physiological conditions, and a small proportion of them occasionally divides to maintain self-renewal abilities and keep balance of the stem cell pool. Notably, under stress or pathological conditions such as hemorrhage or radiation exposure, HSCs are activated and enter the cell cycle in response to external challenges. However, how HSCs integrate external stimuli and respond appropriately requires further elaboration. New technologies, especially emerging single-cell analysis and cell fate tracing techniques, are continuously impacting the traditional understanding of the hierarchical model of hematopoiesis [4]. Multiple models of HSC development have been proposed to provide a greater understanding of hematopoiesis [5,6,7,8]. The consensus is that a complex network exists to orchestrate the hematopoietic process, meanwhile several proinflammatory signals have been demonstrated to be critical regulators of HSC development in the past few years. In this context, a deep understanding of how external cues such as infection, tissue damage, and physical stimuli impact HSC fate would be of great biological significance.

Ever since the term inflammasome was originally put forward in 2002, many studies have indicated that the inflammasome is an intracellular protein complex. The inflammasome is generally formed by a pattern recognition receptor (PRR), apoptosis-associated speck-like protein (ASC) and the inflammatory cysteine protease caspase-1 [9]. These supramolecular structures can be assembled in immune cell cytoplasm, resulting in systemic immune responses and inflammation. As requisite mediators of the innate immune response, inflammasomes serve as multiprotein scaffolds with two main functions: inflammatory reactions and systematic cell death [10,11,12]. Activation of inflammasomes promotes the maturation of the accumulating proinflammatory cytokines interleukin-1β (IL-1β) and interleukin-18 (IL-18) through caspase-1 cleavage. IL-1β can also stimulate the release of other cytokines for example IL-1α, tumor necrosis factor (TNF)-α and IL-6 impacting the function of immune cells [13]. A cascade of downstream events originating from MyD88 recruitment by IL-1R or IL-18R will result in the activation of important signaling proteins and transcription factors, such as NF-κB, regulating inflammation [14]. Inflammasome activation also induces gasdermin D (GSDMD) cleavage by caspase-1, GSDMD as a key pyroptotic substrate of inflammatory caspases, the N-terminal of GSDMD fragment oligomerizes and inserts into the plasma membrane which induces pyroptosis [10,15,16,17]. Pyroptosis can be defined as a lytic form of programed cell death in response to external stimuli or host-derived danger signals, and it distinct from apoptosis by releasing inflammatory compounds into the extracellular space after cell swelling and membrane rupture [18].

Inflammation is a protective immune response that maintains homeostasis and involves various pathological processes, such as pathogen infection and tissue/organ damage. Several kinds of immune cells originating from HSCs constitute the foundation of the inflammatory response, and these cells are continuously replenished during infection to a certain extent [19]. Thus, understanding how HSCs respond to pathological alterations during inflammation is a meaningful research focus. Recent studies have also indicated that inflammasome activation during the inflammatory response plays an essential role in balancing multiple stages of hematopoietic homeostasis [20,21,22,23,24,25,26]. Both up- and down-regulation of inflammasome proteins can lead to a general inclination in homeostasis, suggesting that inflammasome activation may be required to carefully preserve hematopoiesis [21,27,28,29,30,31].

In this review, we will mainly discuss the impact of inflammasome activation on hematopoietic homeostasis. Previous studies have shown that the NLRP3 inflammasome is involved in both normal and malignant hematopoiesis, and the activation of other subtypes of inflammasomes has also been explored in the past few years [20,22,23,29,32]. Furthermore, the cellular and molecular mechanisms of purinergic receptor integration of extracellular cues to activate inflammasomes will be described. The therapeutic potential of targeting the inflammasome and its components in certain hematopoietic diseases through pharmacological or genetic strategies will also be discussed.

## 2. Relationship Between Inflammasomes and Hematopoiesis

The inflammasome assembles in response to danger signals, and inflammasome activation leads to inflammatory responses. There are two main types of signaling pathways involved in inflammasome activation: the canonical signaling pathway and the noncanonical signaling pathway. Numerous studies have suggested that the canonical signaling pathway, which was the first pathway discovered, plays a pivotal role in inflammatory responses and the pathogenesis of various inflammatory diseases [33,34]. There have only been a limited number of studies investigating the role of the noncanonical pathway in inflammatory responses, which mainly include murine caspase-11 activation and human caspase-4 and caspase-5 activation [35,36]. The canonical inflammasome pathway includes a group of nucleotide-binding oligomerization domain (NOD)-like receptors (NLRs) that is mainly composed of NLRP1, NLRP2, NLRP3, NLRP4, NLRP6, NLRP12, and absent in melanoma 2 (AIM2) [37]. Classically, canonical inflammasome activation is initiated by two kinds of signals and regulated at both the transcriptional and posttranslational levels. “Signal 1” is the priming signal and is associated with activation of the TLR/NF-κB pathway or mitochondrial-derived reactive oxygen species (ROS) that activate the TLR4/MyD88 signaling pathway. “Signal 2” can be induced by various stimuli, including pathogen-associated molecular patterns (PAMPs), damage-associated molecular patterns (DAMPs), adenosine triphosphate (ATP), and uric acid crystals [29,38].

The hematopoietic system is classically divided into two major branches during the early stage of hematopoiesis: the myeloid lineage and lymphoid lineage; one prominent function of the myeloid lineage is to establish innate immunity [4,8,39]. Myeloid lineage cells in the circulation mainly include various innate immune cells, such as monocytes, neutrophils, eosinophils, basophils, and dendritic cells (DCs). These cells provide general defense against external pathogens and facilitate adaptive immune responses when they encounter various stimuli. Among lymphoid lineage cells, B cells and T cells participate in adaptive immunity [39,40]. Rapid adaptation of the hematopoietic stem/progenitor cell (HSPC) response to severe bacterial infection leads to peripheral blood (PB) neutrophilia and is defined as emergency granulopoiesis [20,23]. Such responses meet the increasing demand for the generation of immune cells, thus contributing to the chronicity of inflammatory diseases [41,42]. The significant role of inflammasomes in mediating the myeloid lineage and lymphoid lineage has been proven, especially in chronic metabolic diseases. Furthermore, the adaptation of HSPCs to inflammation has also been proven to be a critical event during the host response to infection, microenvironmental stress, or sterile inflammation [43].

### 2.1. Inflammasomes and HSPC Maintenance

BM has hematopoietic functions throughout life and mainly maintains a stable HSC pool. Accumulating evidence has suggested that the inflammasome is involved in different stages of hematopoiesis, and several kinds of inflammasome components have been demonstrated to impact HSPC maintenance [44,45].

**Figure 1:**
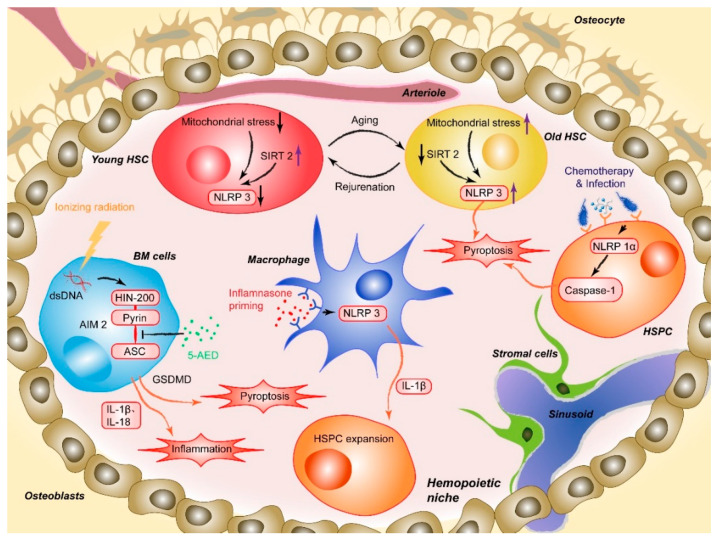
AIM2 is activated after the HIN200 domain senses cytoplasmic double-stranded DNA (dsDNA) caused by ionizing irradiation and the pyrin domain recruits the adapter protein ASC, which induces HSPC death. 5-AED inhibited radiation-induced AIM2 inflammasome activation by decreasing the interaction between AIM2 and ASC. Mitochondrial stress initiates aberrant activation of the NLRP3, and SIRT2 activation inhibits the activation of the NLRP3 inflammasome in HSCs, which suggests a method for reversing aging HSCs. NLRP1α inflammasome activation through chemotherapy or viral infection induces HSPC pyroptosis, resulting in cytopenia, including leukopenia and BM hypoplasia, in the absence of IL-1β-driven inflammation. NLRP3 inflammasome-mediated IL-1β signaling in macrophages drives HSPC production in response to metabolic activity.

Masters and colleagues first reported the pathophysiological effect of NLRP1 inflammasome activation on HSPCs [20]. The researchers noted that activation of the NLRP1α inflammasome in murine HSPCs induces a deadly systemic inflammatory disease that was driven by caspase-1 and IL-1β, independent of ASC and enhanced by IL-18 [20]. Activation of the NLRP1α inflammasome also triggers pyroptosis in HSPCs, resulting in leukopenia and BM hypoplasia, even in the absence of IL-1β-driven inflammation [20]. NLRP1α-deficient mice exhibit enhanced recovery from chemotherapy or viral infection, suggesting that the deletion of NLRP1α effectively increases the resistance of HSPCs to hematopoietic stresses [20]. This finding provides a potential intervention strategy for treating infection-induced cytopenias through which the competence of HSPCs under hematopoietic stress can be protected by removing or pharmacologically inhibiting NLRP1 inflammasome activation (Figure 1) [20]. Later, Hu et al. identified AIM2, another type of inflammasome that mediates HSPC death after whole-body irradiation in mice. The AIM2 inflammasome recruits ASC through its pyrin domain and forms an inflammasome to activate the canonical pathway in a cell-autonomous manner. AIM2-deficient mice are exempted from irradiation-induced hematopoietic failure, as AIM2 acts as a double-stranded DNA sensor that mediates the molecular mechanism of hematopoietic cell death in response to radiation-induced DNA damage (Figure 1). It has also been proposed that inhibiting AIM2 inflammasome activation (e.g., via MCC950, also known as CRID3) is a strategy to treat patients exposed to ionizing radiation due to events such as nuclear reactor leaks or radiotherapy [32].

Moreover, activation of some lineage-specific transcription factors is imperative for mediating the response of HSPCs to infection or sterile inflammation [46]. Metabolic activity has been described as a critical factor regulating stem cell fate decisions, proliferation, and differentiation. In a recently published paper, Frame et al. demonstrated that NLRP3 inflammasome-mediated IL-1β signaling within macrophages in response to metabolic alterations could be an enhancing factor to drive HSPC production in a zebrafish model (Figure 1). The inflammasome serves as a metabolic sensor to trigger IL-1β production and expand developing HSPCs, while up- or down-regulation of the NLRP3 inflammasome accordantly changes the production of HSPCs inside zebrafish embryos or human HSPC cultures [24]. Changes in external conditions can also impact the adaptation of HSPCs. For example, BM cells and hematopoiesis can be severely affected by high-dose radiotherapy [47,48,49]. The process involves a series of cellular and molecular changes, such as the depletion of hematopoietic cells, proinflammatory cytokine and chemokine release, activation and destruction of peripheral immune cells, and DNA damage [49,50]. Accumulating evidence has shown that inflammasome activation plays an important role in mediating radiation-induced cell and tissue damage [51,52]. Specifically, various recruited cells during radiation-induced damage, especially macrophages, are activated to undergo pyroptosis through the NLRP3-caspase-1 axis. Knockout of NLRP3 protected mice from radiation-induced macrophage pyroptosis by suppressing caspase-1 activation [51]. Moreover, given the abovementioned radiation-induced upregulation of the AIM2 inflammasome, the latest research by Wu et al. suggested that 5-androstenediol (5-androstene-3β-17β-diol, 5-AED), a natural steroid hormone produced by the adrenal cortex, could markedly attenuate irradiation-induced AIM2 inflammasome activation, promoting the survival of mice. Subcutaneous administration of 5-AED enhances the recovery of the hematopoietic system and decreases tissue damage by promoting NF-κB signaling and inhibiting inflammasome-mediated pyroptosis possibly by disrupting the interaction between AIM2 and ASC (Figure 1) [53]. A study by Li et al. illustrated that 2-Gy irradiation increased the protein expression levels of NLRP3 in THP-1 cells and elevated ROS levels [54].

While these studies documented the role of inflammasome activation in inhibiting hematopoiesis, activation of another inflammasome subtype also seems to have some positive effects on hematopoiesis. Linz et al. demonstrated that NLRP12 profoundly impacts hematopoietic recovery by suppressing TNF signaling in vivo during emergency hematopoiesis induced by the combination of radiation exposure and thermal injury. As a checkpoint of TNF signaling, the NLRP12 inflammasome functionally limits TNF-induced HSPC apoptosis, and it has been proven that inflammation in the absence of NLRP12 participation leads to HSPC apoptosis, as well as defective peripheral immune reconstitution. In addition, myelopoiesis and immune cell reconstitution are also accelerated by NLRP12 overexpression [23]. Du et al. illustrated that chronic DNA damage upregulates the NLRP12 inflammasome in HSPCs from Fanca^−/−^ mice. In a newly published paper, the researchers further investigated the essential role of NLRP12 in HSC maintenance and found that persistent DNA damage-induced NLRP12 improves HSC function in both mouse and human models of DNA repair deficiency (Fanca^−/−^ mice). Functionally, knockdown of NLRP12 exacerbates the repopulation defect in Fanca^−/−^ HSCs, and overexpression of NLRP12 substantially improves the long-term repopulating function of Fanca^−/−^ HSCs, suggesting a potential genetic or pharmacological strategy to target the NLRP12 inflammasome to obtain therapeutic effects [22,55]. In fact, the lineage contribution of HSPCs in hematopoiesis is no less than that of HSCs, and HSPCs serve as active players in the innate immune response to systemic stimuli, including DNA damage. We hypothesize that HSPCs exert a similar function in Fanca^−/−^ mice as HSCs.

Collectively, the abovementioned effects of different inflammasomes on the maintenance of hematopoiesis still need to be further addressed regardless of physiology or pathology. Consistent with the existing research, we believe there is an interactive transcription network through which signals converge and subsequently regulate inflammasome activity to maintain steady-state hematopoiesis [56,57,58,59,60,61].

### 2.2. Inflammasomes and HSPC Differentiation

Differentiation refers to the process by which progenitor cells develop the appearance of mature PB cells, and the construction of a hierarchical system has gained much attention. Initial lineage priming of the differentiation process is strictly managed by gene-expression modules regulated by lineage-specific transcription factors [62,63]. Some studies have shown that the inflammasome and its components also play a decisive role in HSPC differentiation [57,58,64].

Earlier studies have proven that caspase activation is closely related to the differentiation of several myeloid lineages; as a critical transcription factor, GATA-1 controls erythroid differentiation and pro-platelet formation and maturation [65]. GATA-1 is preferentially localized in the nucleus through an elaborate balance achieved by the interaction between caspase-3 and chaperone HSP70 to prevent cleavage [66]. Under conditions in there is an acute need for platelets, caspase-3 can be activated in response to IL-1α, thus promoting the formation of platelets. IL-1β is usually secreted by monocytes in response to lipopolysaccharide. through an inflammasome activation-dependent pathway. A recent report from the Tyrkalska group supported this view, indicating that inflammasomes participate in erythroid/myeloid cell fate decisions and that terminal erythroid differentiation in chronic inflammatory diseases eventually contributes to hematopoietic bias [21,66]. Pharmacological inhibition of the inflammasome ameliorated neutrophilic inflammation and anemia in zebrafish disease models. GATA-1 is increased in inflammasome-deficient larvae and is responsible for facilitating erythropoiesis and inhibiting myelopoiesis. Interestingly, inflammasome inhibition did not affect the granulocyte-monocyte myeloid transcription factor PU.1 (SPI1) level, indicating that there are some indirect effects left to explore. These results show that the inflammasome plays an essential role in the pathogenesis of neutrophilia and anemia during chronic inflammatory diseases, suggesting a pharmacological target for therapeutic interventions [21].

Acute myeloid leukemia (AML) is characterized by the blockade of hematopoietic differentiation and cell death, and interesting work from the Jost laboratory demonstrated that receptor-interacting protein kinase 3 (RIPK3) promotes the differentiation of leukemia-initiating cells by activating the inflammasome. RIPK3 suppresses malignant myeloproliferation by activating the inflammasome, thus promoting differentiation and cell death, and RIPK3 expression is often reduced in primary de novo AML to prevent leukemia-initiating cells from dying [67].

### 2.3. Inflammasomes and Aging-Associated Hematopoiesis

Aging is an unavoidable consequence of life, and enhanced myelopoiesis is a hallmark of BM aging and impaired lymphopoiesis, which are mainly caused by myeloid-biased HSPC proliferation and differentiation. This alteration in hematopoiesis is sometimes referred to in the literature as inflamm-aging, which is the chronic, low-grade sterile inflammation that is present in advanced age and manifests some relevant clinical symptoms.

The Dixit group first demonstrated the role of the NLRP3 inflammasome in promoting age-related thymic atrophy and immune senescence. The researchers found that deletion of the inflammasome components NLRP3 and ASC significantly increased the number of cortical thymic epithelial cells and T cell progenitors, which reduced aging-related thymic atrophy. The deletion also accelerated T cell reconstitution and immune recovery in middle-aged animals, suggesting an NLRP3 inflammasome-dependent mechanism through thymic caspase-1 activation mediates this process [68,69]. Recently, another study showed that the NLRP3 inflammasome was aberrantly activated in HSCs during physiological aging. This activation was mainly mediated by mitochondrial stress and SIRT2 inactivation, contributing to the functional decline in aging HSCs. As a cytosolic NAD^+^-dependent deacetylase, SIRT2 is required for HSC maintenance and regenerative capacity during senescence by suppressing the activation of the NLRP3 inflammasome in HSCs [69,70,71].

Luo et al. have demonstrated that SIRT2 regulates the functional deterioration of HSCs in aging models by repressing the NLRP3 inflammasome activation, which SIRT2 activation, NLRP3 inflammasome inactivation or caspase-1 inactivation improves the maintenance and regenerative capacity of aged HSCs [72]. Functionally, overexpression of SIRT2 can increase the maintenance and regenerative capacity of aged HSCs, which did not significantly influence young HSCs. Thus, these results indicate a potential SIRT2-NLRP3-caspase-1 axis in which the function of senescent hematopoietic and immune cells can be maintained or even rejuvenated. In contrast, in the previously described study, aging-related, persistent DNA damage-induced NLRP12 expression improved HSC function in both mouse and human models of DNA repair deficiency (Fanca^−/−^ mice). The authors found that the depletion of NLRP12 in aged HSCs compromised their self-renewal and hematopoietic recovery capacities, suggesting that pharmacological activation of NLRP12 may have therapeutic value in enhancing the function of aged HSCs [22,55].

## 3. Extracellular Signals Activate the Inflammasome through Purinergic Receptors to Mediate HSPC Trafficking

How HSCs in the BM sense changes in the body and respond appropriately to external physiological or pathological stimuli has not been well elucidated thus far. Ratajczak’s group examined this process by implementing a series of experiments focusing on HSPC mobilization, homing, and engraftment [25,29,73,74,75,76,77,78,79]. Their results and conclusions have been published in some of the comprehensive and elegant articles that we mentioned previously. As an essential participant in innate immunity, the inflammasome detects and senses various sterile or infectious stimuli that the cells encounter and then mediates cellular responses. Activation of the inflammasome acts as a neutralizer inside innate immune cells to not only induce an inflammatory cascade but also connect the complement cascade (ComC) with the HSPC lifecycle. Here, we will briefly delineate these steps, including the activation of purinergic receptors by exclusive extracellular mobilizing factors, active inflammasome mediation of the ComC via the mannan-binding lectin (MBL) pathway, and the accompanying egress of HSCs into the PB.

### 3.1. Structure, Distribution, and Activation of Purinergic Receptors

Purinergic receptors, also known as purinoceptors, are present in multicellular organisms that utilize purines and pyrimidines as signaling molecules to mediate cellular responses. Purinergic receptors are widely expressed throughout the body and are mainly used for cellular communication, genetic information transfer, and energy metabolism. Purinergic receptors are categorized into two classes based on their activation mechanism: P1 (preferentially activated by adenosine (ADO)) and P2 (activated by various nucleotides). P1 receptors are G protein-coupled cell-surface receptors that can be further divided into four subtypes: A1, A2A, A2B, and A3 [80]. A1 and A3 receptors are coupled to G_i_, Gq, and Go proteins, and stimulation of these receptors can also cause the release of calcium ions from cells [81]. A2A and A2B receptors are linked to Gs or Golf and are stimulated by adenylyl cyclase, while the A2B receptor can activate phospholipase C through Gq in addition to Gs [82]. Furthermore, all ADO receptors are coupled to mitogen-activated protein kinase (MAPK) pathways, such as extracellular signal-regulated kinase 1 (ERK1), extracellular signal-regulated kinase 2 (ERK2), p38 MAPK, and JUNN-terminal kinase [80]. P2 receptors are activated by numerous nucleotides, and the nucleotide receptor family is composed of two subtypes: ligand-gated ionotropic P2X receptors (P2XRs) and G protein-coupled metabotropic P2Y receptors (P2YRs). P2 receptor-mediated events frequently occur since their activation by extracellular nucleotides can occur during responses to tissue injury, infection, shear stress, or cell death [83]. There are ample opportunities to modulate P2 receptor-mediated events because extracellular nucleotides are released in response to tissue injury, infection, shear stress, and cell death [83]. There are fifteen P2 receptors in total, and the P2X and P2Y subtypes include seven (P2X1–7) and eight (P2Y1, 2, 4, 6, 11–14) members, respectively, based on their sequence, agonist selectivity, and membrane topology [84].

ATP, ADP, UTP, UDP, and ADO are the main ligands of purinergic receptors and are synthesized intracellularly and released via exocytosis, plasma membrane proteins (Panx1, P2X7), plasma membrane vesicles, and stress. Immune cells, hematopoietic cells, neuronal cells, cancer cells, and endothelial cells are all known to release nucleotides, particularly ATP, into the extracellular space [85,86,87,88]. The process is essential for innate and acquired immune responses, hematopoiesis, neuronal transmission, or tumorigenesis. ATP and ADP are also hydrolyzed by the ecto-nucleotidases CD39 and CD73 to produce the P1 receptor agonist ADO extracellularly. In fact, extracellular ATP (eATP) exerts primarily proinflammatory effects, while ADO is a potent immunosuppressant [88,89,90]. Extracellular ADO can also be transported into the cytoplasm by equilibrative nucleoside transporters 1 and 2 (ENT1 and ENT2) or it can also be degraded to inosine by ADO deaminase (ADA)/CD26 extracellularly [84]. Extracellular ATP mainly activates P2XRs, and the P2YR subtypes are seven-membrane-spanning G protein-coupled receptors that are mainly activated by nucleotides. P2Y1, P2Y2, and P2Y11 can be activated by ATP, while UTP and UDP mainly activate P2Y2, P2Y4, and P2Y6, and only P2Y14 is activated by the nucleotide sugar UDP-glucose [84]. Depending on tissue type, the involvement of P2XRs and P2YRs triggers various responses, including cell proliferation or differentiation, necrotic cell death or apoptosis, secretory exocytosis, chemotaxis, ROS generation, and cytokine release.

### 3.2. HSPC Trafficking and Inflammasomes

The BM is a dynamic organ that responds to physical and pathological signals. Recirculation of HSPCs between the BM and PB is a crucial process that we simplify with the term “trafficking.” Interactions between HSPC-expressed receptors and their respective stroma-expressed ligands mainly determine the anchoring ability of HSPCs within the BM [89]. Specifically, studies have shown that CXCR4 and very late antigen 4 receptor (VLA-4, also known as α4β1-integrin), which are expressed on the cell surface of HSPCs, are retained in the BM niche by interactions with their respective ligands, including α-chemokine stromal cell-derived factor 1 (SDF-1, also known as CXCL12) and vascular adhesion molecule 1 (VCAM-1, also known as CD106), which are mainly expressed by cells inside the BM microenvironment [91].

Mobilization is a detachment step by which HSPCs migrate from the BM to PB and involves the upregulation of proteolytic enzymes expressed either by stromal elements or HSPCs, and the activation of these enzymes loosens the connections between the BM niche and HSPCs by influencing the SDF-1-CXCR4 or VCAM-1-VLA-4 axis [92]. Moreover, complete homing and engraftment of HSPCs in BM are the major determining factors in the success of transplantation. As a multistep process orchestrated by the interplay between adhesion molecules, cytokines, and regulatory cofactors, HSPCs are drawn to organs mainly by the chemotactic activity of SDF-1, sphingosine-1-phosphate (S1P), ceramide-1-phosphate (C1P), and eATP [74,93]. Deficiencies in these factors in the BM microenvironment or a lack of corresponding receptors on the surface of HSPCs results in impaired homing and engraftment of transplanted HSPCs to BM niches. Based on this mechanism, a viable method in which HSPCs can be mobilized into the PB by antagonists of CXCR4 (Plerixafor, also known as AMD3100) or inhibitors VLA-4 (BIO5192) was developed. Cell membranes containing combinations of glycosphingolipids and protein receptors are organized into a glycoprotein microdomain known as a lipid raft and play essential roles in orchestrating the migration of HSPCs toward increased concentrations of chemotactic factors. Notably, both CXCR4 and VLA-4 receptors are associated with membrane lipid rafts located on the HSPC surface. The integrity and stabilization of membrane lipid rafts are mainly determined by glycosylphosphatidylinositol (GPI) anchor proteins (GPI-APs), which are susceptible to perturbation by the lipolytic enzyme phospholipase C-β2 (PLC-β2), which is released by innate immune cells to attenuate HSPC retention in BM during mobilization [92].

A series of studies by the Ratajczak group indicated a novel mechanism by which the NLRP3 inflammasome plays dual roles in the regulation of HSPC migration toward BM chemoattractants and also responds to myeloablative irradiation of the BM microenvironment to facilitate the homing and engraftment of transplanted cells [94]. The researchers first illustrated that the MBL-initiated ComC and coagulation cascade (CoaC) are involved in triggering the mobilization of HSPCs [95]. ATP is released extracellularly primarily through the pannexin-1 channel and activates the MBL-ComC (CoaC) pathway as a DAMP via purinergic receptors [95,96,97]. The researchers then proposed that the ATP-induced NLRP3 inflammasome acts as a neutralizer that connects purinergic signaling with the activation of the ComC, which is required for the egress of HSPCs from the BM into the PB [98]. In fact, proper NLRP3 inflammasome expression in HSPCs is also required for the migration of HSPCs in response to BM-expressed homing factors [94]. Activation of the NLRP3 inflammasome in HSPCs promotes the incorporation of CXCR4 into membrane lipid rafts, which enhances the release of eATP in an autocrine/paracrine manner to facilitate the migration of HSPCs in response to an SDF-1 gradient [94]. NLRP3 inflammasome deficiencies within the BM of transplantation recipients induces a negative effect on the homing and engraftment of transplanted HSPCs, which may be due to the reduced mRNA expression of SDF-1 and certain DAMPs responsible for ComC activation [94]. Sustained activation of the NLRP3 inflammasome during mobilization induces the release of ROS and mitochondrial DNA from eATP-activated cells. These stimuli combine with other DAMP molecules, such as high molecular group box 1 (HGMB-1) and S100 calcium-binding protein A9 (S100a9), and activate the ComC, which is crucial for the egress of HSPCs from the BM into the PB.

Moreover, inflammasomes are activated during sterile inflammation, which induces intracellular caspase-1 to promote the maturation of proinflammatory cytokines, the secretion of IL-1β and IL-18, and pyroptosis [94,98]. In addition to their proinflammatory properties, these two cytokines exert several other pleiotropic effects during hematopoiesis. On the one hand, IL-1β stimulation downregulates p21 as a cell cycle inhibitor, resulting in self-renewal impairment and decreased reconstitution capacity, while during ionizing radiation, IL-1β may protect adult BM HSPCs from cell death and increase their proliferation and differentiation abilities [99,100]. On the other hand, IL-18 is mainly involved in the Th1 paradigm, inducing the production of IFN-γ, modulating early differentiation of hematopoietic cells and consequently enhancing the production of mature cells to act against infection [101]. Interestingly, IL-1β and IL-18 released from innate immune cells during inflammasome activation also increase the egress of these cells from the BM, and both cytokines are strong mobilization mediators [100,102]. The release of active IL-1β and IL-18 from cells can provide molecular evidence for inflammasome activation, and other DAMPs are also released in addition to interleukins. Some cell-secreted DAMPs can be recognized by mannan-binding lectin (MBL), resulting in the activation of the MBL-MASP (MBL-associated serine protease) pathway of ComC activation in the initiation phase of mobilization [77,95]. During inflammasome activation, these cytokines are important endogenous mediators that amplify the mobilization process.

### 3.3. ATP Participates in the Mobilization of HSPCs

Based on the mobilization, homing, and engraftment mechanisms of HSPCs, effective treatments have been successfully developed for numerous malignant and nonmalignant disorders, including leukemia, lymphoma, and selected inherited genetic diseases [103]. Strenuous exercise, tissue injury, and the administration of specific cytokines (granulocyte mobilizing factor, G-CSF) or chemokines (Plerixafor) can induce a cascade of events in the BM microenvironment through sterile inflammation or infection, which activate various innate immune cells, including macrophages and neutrophils. G-CSF or AMD3100 induces the egress of HSPCs into the PB. The activation of innate immune cells (especially Gr-1^+^ leucocytes) in the BM microenvironment releases DAMPs, including ATP, proteolytic and lipolytic enzymes, HGMB-1, S100a9, and ROS [104].

One of the most critical DAMPs released during the initiation phase of mobilization is eATP, which is also an effective activator of the inflammasome [105,106,107]. Conventionally, ATP is a type of fuel for cells, and it is a well-known ubiquitous intracellular molecular energy source [108]. However, when secreted from cells, ATP becomes a crucial signaling molecule in the purinergic signaling network [85,109,110]. ATP and ADO have already been proven to promote the proliferation of HSPCs and the trafficking of granulocytes and monocytes, and these molecules also inhibit the proliferation and migration of leukemic cells [96,111,112,113]. eATP is released from cells via exocytosis or pannexins and connexins in an autocrine and paracrine manner and couples with purinergic receptors on the surface of HSPCs to trigger the egress of HSPCs from the BM into the PB [77,114].

Therefore, eATP activates the NLRP3 inflammasome through the P2X7 and P2X4 receptors, which are highly expressed on the surface of hematopoietic cells. Inflammasome activation potentiates the release of other DAMPs, proteolytic and lipolytic enzymes, and several proinflammatory cytokines and chemokines from innate immune cells. The entire process also involves the activation of intracellular caspase-1 and the release of mature IL-1β and IL18 into the extracellular space [96,115,116]. This activation and release maintain sterile inflammation in the BM microenvironment. ATP in the cytoplasm is directly recognized by MBL, which then activates the MBL-associated serine proteases MASP-1 and MASP-2 to initiate the ComC. In addition, MASP-1 is also involved in the activation of the CoaC by activating prothrombin to thrombin, which interacts with the ComC to affect the mobilization process. The crosstalk between both cascades enhances the activation of the distal part of ComC via the cleavage of C5 into C5a and desArgC5a, and C5aR activates innate immune cells and the NLRP3 inflammasome [74]. In addition to P2X receptors, the role of metabotropic P2Y receptors in the trafficking of HSPCs also requires further examination, since studies have shown that P2Y2, P2Y4, and P2Y6 receptors participate in innate immune responses [117]. Moreover, ADO, a metabolite of ATP, and intracellular heme oxygenase 1 (HO-1) have been proven to be negative regulators of the mobilization process by inhibiting inflammasome activation and the mobilizing effects of ComC-induced C5a [79,118,119].

## 4. Inflammasomes in Hematological Diseases

In the past two decades, a series of studies have shown that chronic inflammation in the tissue microenvironment generates oncogenic mutations and genomic instability, which play vital roles in cancer development and progression. As the main components of the innate immune response, the role of inflammasomes has been shown to be dependent on the type of tumor, and specific inflammasome subtypes are also involved. Activation of the inflammasome induces cell death via pyroptosis and the secretion of IL-1β and IL-18, mainly through the activation and release of caspase-1 from the complex. Moreover, the accumulation of somatic DNA mutations in highly proliferative hematopoietic cells results in aberrant HSPC proliferative advantages, leading to clonal expansion. This somatic mutation-induced clonal hematopoiesis is now relatively common in cancer-free, asymptomatic, and old individuals. The combination of these pathological alterations commonly occurs in the hematopoietic system and ultimately constitutes the basis of several kinds of hematological diseases [14,120].

### 4.1. Myelodysplastic Syndromes (MDSs)

MDSs are myeloid neoplasms that are mainly characterized by the clonal proliferation of HSCs, myelodysplasia, ineffective hematopoiesis, peripheral blood cytopenia, and a high-risk of AML development [121,122]. In the past decade, it has been recognized that aberrant innate immune activation and proinflammatory signaling act as key pathogenic drivers of MDS [123]. Basiorka et al. demonstrated that HSPCs from MDS overexpress inflammasome proteins and exhibit aberrant activation of NLRP3, which activates caspase-1 and generates mature IL-1β and IL-18, eventually causing massive pyroptotic cell death in HSPCs [28]. An excess of the alarmin protein S100A9 in MDS HSPCs and BM plasma activates NADPH oxidase and increases the level of ROS to initiate cation influx, cell swelling, and β-catenin activation. To date, aberrant inflammasome signaling is regarded as a fundamental driver of MDS. In addition, studies have shown that either neutralization of S100A9 or inhibition of inflammasome components prevented pyroptosis, restored the appropriate ratio of hematopoietic progenitors, and improved erythropoiesis in an S1009A transgenic mouse model [28]. Therefore, biomarkers, as well as inflammasome inhibitors, would be valuable in the diagnosis and treatment of MDS and have attracted much attention in the past decade. In the future, more detailed investigations should assess the feasibility of inflammasome inhibitors for treating MDS, as well as suppressing downstream chemokines or effector molecules.

### 4.2. Myeloproliferative Neoplasms (MPNs)

MPNs are a group of clonal hematologic disorders characterized by the overproliferation of abnormal HSPCs [124]. It represents a unique model of the relationship between chronic inflammation and clonal hematopoiesis of a hematologic malignancy. The inflammatory microenvironment of MPN is also related to the continuous release of proinflammatory cytokines, chemokines, and ROS in the hematopoietic stem cell compartment, which might lead to successive development of neoplasms [125,126]. MPNs include three main conditions: polycythemia vera, essential thrombocythemia, and myelofibrosis, which are related to frequent disease-related complications, including thrombosis, hemorrhage, and the transformation to MDS or AML [127,128]. It has been proven that inflammasome-associated chronic inflammation, as a hallmark of cancer development that affects all tumorigenesis stages, including initiation, promotion, malignant conversion, invasion, and metastasis, is driven by MPN neoplastic clones [129,130]. In fact, cancer-related inflammation can be triggered by a series of signals from immune system cells including macrophages, neutrophils, T and B lymphocytes, and NK cells [128].

In the past few years, several studies have shown that some genes involved in inflammasome activation were significantly overexpressed in MPNs, indicating an important role of the inflammasome in the pathogenesis and progression of MPN [129,131,132]. Some studies have indicated a contradictory effect of the inflammasome inside tumors to act as a double-edge sword contributing to the pathogenesis and progression of the neoplasm as well as the maintenance of the tumor microenvironment, and also suppress tumor growth through pyroptosis on the contrary [120,133]. Early studies illustrated that many cytokines, especially IL-1β and IL-18, were released following inflammasome activation and positively promoted the inflammatory process [132,134]. In parallel, direct or indirect dysregulation of JAK2 signaling by somatically acquired mutations has emerged as a central phenotypic driver of classic MPNs. The Tet-inducible JAK2V617F-expressing cell line D9 was used to show that the genes associated with inflammasome activation, including AIM2, CASP1, and IL-1β, were strongly induced by JAK2V617F, and AIM2 is a downstream target of JAK2V617F in D9 cells [131]. However, to date, the functional role of HSPC inflammasome signaling in MPN pathogenesis remains to be explored.

### 4.3. Graft-Versus-Host Disease (GVHD)

GvHD occurs in 30% to 40% allogeneic HSC transplantation (allo-HSCT) patients, has high mortality and has become a significant obstacle to the success of HSC transplantation [135]. GvHD is mediated by donor T cell recognition of host-derived alloantigen expressed on antigen-presenting cells (APCs) and further accelerated by the recipient inflammatory milieu and tissue injury [135]. Tissue damage can lead to the recruitment of other effector cells (e.g., NK cells and PMNs), accelerating tissue injury and resulting in a self-perpetuating state. Such a vicious cycle makes it difficult to control this condition [136,137]. Therefore, a better understanding of the molecular mechanism of GvHD may help to improve the outcome of allo-HSCT patients. Jankovic et al. investigated the role of the NLRP3 inflammasome in regulating the occurrence of GvHD and demonstrated that the NLRP3 inflammasome components NLRP3 and ASC were required for the full manifestation of GvHD. Intestinal commensal bacteria and the DAMP uric acid contribute to NLRP3 inflammasome-mediated IL-1 production after conditioning therapy. Early blockade of IL-1β or genetic knockout of the IL-1 receptor in DCs and T cells improved cell survival after transplantation. Consistently, increased levels of active caspase-1 and IL-1β were found in circulating leukocytes and intestinal lesions of GvHD patients. As the central cytokine that promotes acute GvHD, IL-1β induces allogenic T cells to differentiate into T helper 17 cells, which are a subset of proinflammatory T helper cells that are implicated in autoimmune and inflammatory disorders, further initiating GvHD [138].

One potential method to control GvHD is immunosuppressive cell therapy, and myeloid-derived suppressor cells (MDSCs), which are potent suppressors of alloimmunity, have been defined broadly as myeloid lineage cells with suppressive capacities [139,140]. However, the efficacy of this treatment has limited efficacy in preventing the occurrence of acute GvHD due to inflammasome activation, and donor MDSC infusion only partially ameliorates GvHD lethality. Koehn et al. found that MDSCs transferred to lethally irradiated mice with allogeneic donor HSCs are confronted with an intense inflammatory environment associated with GvHD and then rapidly lose their suppressor functions [141]. In a more recent study, the researchers demonstrated that extracellular ATP is the primary driver of MDSC dysfunction through P2X7 receptor engagement and NLRP3 inflammasome activation [142]. ATP binds to the P2X7 receptor on APCs, which leads to the augmented expression of costimulatory molecules, resulting in robust activation of alloreactive T cells and a severe GvHD phenotype [143]. Importantly, MDSC-specific inhibition of NLRP3 inflammasome activation and IL-1β release rather than systemic inhibition of IL-1β signaling improves the survival of grafts [142]. Betts et al. have discovered that X-box binding protein-1 (XBP-1s) is a relevant therapeutic target to suppress NLRP3 activation in the context of ER stress and prevent GVHD after alloHCT [144]. These findings highlight the critical role of inflammasome activation in both the occurrence and treatment of GvHD.

Recently, a study from China reported that the gut microbial metabolite choline-metabolized trimethylamine N-oxide exacerbates GvHD by inducing M1 macrophage polarization, and the underlying mechanism also involves NLRP3 inflammasome activation [145]. Therefore, further understanding the activation mechanism of the inflammasome in different types of cells and their roles in GvHD will help to effectively prevent and treat GvHD in the clinic. Interestingly, there is evidence that GvHD induction is dependent on functional miR-155 in DCs in allo-HSCT recipients [146]. MiR-155 deficiency in mice resulted in reduced serum levels of proinflammatory cytokines, reduced ATP-mediated cell migration, and decreased inflammasome activation and IL-1β production [147]. This finding suggests a way to use miRNA antagomirs to inhibit proinflammatory effects to reduce this complication of allo-HSCT.

## 5. Conclusions

In the past few decades, further studies of the inflammasome family have led to a better understanding of the pleiotropic effects of inflammasomes on the hematopoietic system. Beginning with the innate immune system, chronic metabolic diseases, and cancer progression, as well as immune cells and upstream lineages, we can definitely expect new and exciting findings regarding the functions of different types of inflammasomes, such as maintenance, expansion, differentiation, trafficking, aging, or the malignant transformation of diverse stem cell lifecycles. These knowledges and the confirmation of relevant targets undoubtedly have potential clinical implications. Here we list a comprehensive table which contains some potential therapies through targeting inflammasomes in the hematopoietic system (Table 1).

Some of the topics that might need to be addressed are the physiological and pathological functions of other inflammasome subtypes, in addition to the most concerning NLRP3 inflammasome, and the mutual effects of these subtypes on pro- and anti-inflammatory processes. Considering the contribution of inflammasomes to hematopoietic homeostasis and cancer progression, more investigations can be performed on inflammasome inhibitors, which may help to provide promising therapeutic and prevention strategies.

## Figures and Tables

**Table 1:** Therapeutic potential of targeting the inflammasome.

Therapeutic Potential	Description	Targets	Stage	Refs.
Pharmacologically optimizing the trafficking	NLRP3 inflammasome is expressed in HSPCs, and purinergic signaling-NLRP3 inflammasome-ComC axis plays an essential role in the HSPC mobilization process. NLRP3 inflammasome is also activated in HSPCs harvested for transplantation, which plays a crucial role during homing and engraftment of HSPCs.	NLRP3	Preclinical	[29]
Protection from GvHD	NLRP3 inflammasome-mediated innate immune plays a critical role in the GvHD initiation. The Nlrp3 and inflammasome components Asc are crucial for the manifestation of GvHD, and early blockade of IL-1β signaling in dendritic cells and T cells improved survival. Loss of NLRP3 function alleviates murine hepatic GvHD.	NLRP3	Preclinical	[138,140,143]
Radiation protection	Radiation induces NLRP3 inflammasome activation and pyroptosis in bone marrow-derived macrophages (BMDMs). AIM2 inflammasome is activated in bone marrow cells in response to double-strand DNA breaks caused by ionizing radiation. NLRP12 has a profound impact on hematopoietic recovery during radiation by serving as a checkpoint of TNF signaling and preventing hematopoietic apoptosis.	NLRP3 AIM2 NLRP12	potential	[23,32,51,52,53,54]
Athero-protective activity	Cholesterol crystal uptake and rupture of macrophage lysosomes can both trigger NLRP3 inflammasome activation. Studies also proved that IL-1β is a driver of cardiovascular disease. Limiting IL-1β signaling alone can significantly protect against heart disease in patients. TET2-deficient macrophages exhibited an increase in NLRP3 inflammasome–mediated IL-1β secretion.	NLRP3	Preclinical	[31,48,148,149]
Treatment of severe acute respiratory syndrome–related coronavirus 2 (SARS-CoV-2) and coronavirus disease 2019 (COVID-19)	SARS-CoV-2 can be recognized by RNA-sensing pattern recognition receptors and activates inflammasomes, and triggers pyroptosis in immune cells. Then cytokines such as IL-1β and IL-6 are elevated in the sera of COVID-19 patients (cytokines storm) and cause acute respiratory distress syndrome (ARDS) etc., injuries.	NLRP3	potential	[9,150,151]

## Abbreviations

5-AED	5-androstenediol
AIM2	absent in melanoma 2
AML	acute myeloid leukemia
ADO	adenosine
ATP	adenosine triphosphate
allo-HSCT	allogeneic HSC transplantation
ASC	apoptosis-associated speck-like protein
BM	bone marrow
C1P	ceramide-1-phosphate
CoaC	coagulation cascade
ComC	complement cascade
DAMPs	damage-associated molecular pattern
DC	dendritic cell
ENT	equilibrative nucleoside transporter
ERK	extracellular signal-regulated kinase
GSDMD	gasdermin D
GPI-A	glycosylphosphatidylinositol anchor
GVHD	graft versus host disease
G-CSF	granulocyte colony-stimulating factor
HSC	hematopoietic stem cell
HO-1	heme oxygenase 1
HGMB-1	high molecular group box 1
IL	interleukin
MBL	manna-binding lectin
MSC	mesenchymal stromal cell
MAPK	mitogen activated protein kinase
MDS	myelodysplastic syndromes
MDSC	myeloid-derived suppressor cell
MPNs	myeloproliferative neoplasm
NLR	nucleotide oligomerization domain-like receptor
PAMP	pathogen-associated molecular pattern
PB	peripheral blood
PLC-β2	phospholipase C-β2
ROS	reactive oxygen species
RIPK3	receptor-interacting protein kinase 3
S100a9	s100 calcium-binding protein A9
S1P	sphingosine-1-phosphate
SDF-1	stromal cell-derived factor 1
VCAM-1	vascular adhesion molecule 1
VLA-4	very late antigen 4 receptor
